# Effect of Addition of Mango Seed Extract on Storage Stability of Chevon Meatballs at Refrigeration Temperature

**DOI:** 10.3390/foods13050676

**Published:** 2024-02-23

**Authors:** Pramila Umaraw, Veer Pal Singh, Akhilesh K. Verma

**Affiliations:** Department of Livestock Products Technology, College of Veterinary and Animal Sciences, Sardar Vallabhbhai Patel University of Agriculture and Technology, Meerut 250 110, Uttar Pradesh, India; pramila1303@gmail.com (P.U.); dr.vplpt@gmail.com (V.P.S.)

**Keywords:** meatballs, antioxidant activity, MSE, lipid oxidation, microbial quality, sensory

## Abstract

In this study, the addition of mango seed extract (MSE) in goat meatballs was assessed. The efficacy of three different levels of MSE extract, namely T1 = (2.5 mL/100 g of meat emulsion *v*/*w*), T2 = (5.0 mL/100 g of meat emulsion *v*/*w*), T3 = (7.5 mL/100 g of meat emulsion *v*/*w*), and T0 (control without mango seed extract), was conducted for evaluation of changes in water activity (a_W_), pH, total phenolic compounds, DPPH, peroxide value, TBARS, microbial quality, and sensory attributes of the goat meatballs stored at refrigerated temperature (4 ± 1 °C). Incorporation of the mango seed extract T3 (7.5 mL/100 g) showed that it can potentially better maintain change in pH and water activity. Total phenolic and DPPH activity decreased significantly (P0.05) among all samples throughout storage; however, the highest value was noted for T3 among all samples. The MSE-added goat meatballs (T3) group had lower significant (*p* < 0.05) peroxide values than the other samples. The T3 sample added with MSE exhibited significant (*p* < 0.05) lower TBRAS values as compared to other treatments. Comparatively lower microbial proliferation and better sensory attributes were maintained among the treated groups during the entire storage time. The results show that the inclusion of MSE extract T3 (7.5 mL/100 g) is a promising natural antioxidant that can maintain a better quality of goat meatballs at refrigerated temperature (4 ± 1 °C) under aerobic packaging conditions.

## 1. Introduction

Goat meat (chevon) is the choicest meat in India due to its unique flavor while also being free from any religious taboo [1]. Higher moisture content and good nutrient quality of meat provide an excellent medium for the proliferation of microorganisms as well as the oxidative deterioration of unsaturated fatty acids making it prone to spoilage [2]. Meat products contain unsaturated fatty acids, and therefore are liable to lipid rancidity and lead to the formation of compounds that have a negative impact on the nutritional quality, taste, and aroma of products. Rancidity and microbial deterioration are the major causes of the formation of free radicals, which have been associated with changes in biological reactions within the living cell and eventually affect consumer health. In the meat industry, products are generally prepared with the addition of synthetic preservatives to enhance their shelf life under regulatory guidelines that limit the use of these synthetic preservatives in meat in low concentrations. Various artificial preservatives are used for the preservation of meat products, but these synthetic compounds have been associated with the occurrence of various lifestyle diseases with long-term consumption [3]. With increasing consumer awareness, the health concern quest for alternative preservatives has taken a leap over the past few decades. Natural, plant-based or plant-derived preservatives are being widely explored to replace synthetic preservatives. Moreover, with increased awareness among consumers and the change in the consumption pattern, the demand for natural, “preservative-free” products during the last few decades has led the meat processing industry to consider the inclusion of natural preservatives in processed meat products. Recently, efforts have been made to utilize various plant parts, herbs, and spices in various forms such as powder, extract, and essential oils to extend the shelf life of the following products: fresh meat, meat emulsion, and/or meat products such as coriander extract with BHT in chicken patties for the reduction in lipid oxidation [4]; mushroom extract on chevon nuggets for evaluation of antioxidant and antimicrobial efficacy [2]; synergistic effects of plant extract and essential oils in chicken and pork products for the extension of shelf life [5]; betel leaf extract as an antioxidant in beef sausages [6]; mango peel extract on ground beef [7]. However, mango seed kernel as a natural preservative has not been explored much in meat products.

The fruit industry discards huge amounts of mango seeds yearly that contain various beneficial photoactive compounds. Therefore, various phyto-preservatives have been evaluated as natural preservatives from plants. Extracts obtained from plants rich with flavonoids, polyphenols, and other bioactive compounds have been fruitfully used to slow down the fat and protein oxidation of meat and meat products during storage. Mango seed extract contains numerous phyto-chemical compounds like gallic acid, ellagic acid, cinnamic acids, tannins, ferulic acid, and mangiferin that show antioxidant and antimicrobial activity [8,9,10]. Studies on the preservative aspect of an extract of mango seed kernel (MSE) in food systems such as meat products have not been extensively investigated in meat products. Therefore, this study was undertaken to contemplate the effect of the addition of MSE on the quality characteristics of goat meatballs during refrigerated storage.

## 2. Material and Methods

### 2.1. Materials

Chevon meat was purchased from the local market Modipuram, Meerut, U.P. and brought to the Department of Livestock Products Technology under chilled conditions. Ripened mango was obtained from a local retailer and its seed was collected after removal of mango pulp. Collected seed was cleaned with water and sundried. After that, the hard covering of the seed was removed and the mango seed kernel was collected followed by drying in a hot air oven for 4 h. The dried MSE was ground and strained to collect as a fine powder. Other ingredients as listed in Table 1 were purchased from the local market and the analytical regents/media used in this experiment were procured from SRL and Hi-media, etc.

### 2.2. Preparation of Extract and Meatballs

In total, 25 g mango seed kernel (MSE) powder was dissolved in 250 mL of sterile distilled water. The MSE extract was prepared as per the method followed by Umaraw et al. [11]. Deboned chevon was cut into small pieces and minced twice (6 and 4 mm) with a meat mincer. Mango seed kernel powder, common salt, chilled water, egg yolk liquid, tri-sodium polyphosphate, nitrite, textured soya flour, refined vegetable oil, condiment and spice mixture were added to the weighed meat as per the formulation (Table 1). Four groups of chevon meat emulsion were prepared with varying levels of mango seed kernel extract on the basis of preliminary trials viz. T1 = (2.5 mL/100 g of meat emulsion *v*/*w*), T2 = (5.0 mL/100 g of meat emulsion *v*/*w*), T3 = (7.5 mL/100 g of meat emulsion *v*/*w*) and T0 (without mango seed extract). Mango seed kernel extract concentration was prepared as 160 mg/mL. Meat emulsion for the preparation of chevon meatballs was prepared as per the procedure described by Verma et al. [2]. After the formation of the desired emulsion, meatballs were prepared manually. Prepared chevon meatballs were stored at refrigerated temperature (4 ± 1 °C) for 15 days and analysis of physico-chemical, antioxidant activity, lipid oxidation, microbial quality, and sensory attributes was conducted at 3-day intervals.

### 2.3. pH and Water Activity

The pH of goat meatballs was recorded by digital pH meter (ESICO, Model-1012, Biogen, Meerut, India) following the methodology given by Trout et al. [12]. A 5 g chevon meatball was homogenized with 45 mL of distilled water for the estimation of pH. Water activity (a_W_) of the chevon meatball was recorded with a Novasina water activity meter (LabSwift-aw, Lachen, Switzerland).

### 2.4. Antioxidant Activity

In total, 10 g of meatballs was weighed and homogenized with 40 mL of methanol and ethanol (1:1) for three minutes. Afterward, this homogenised mixture was sieved through Whatman filter paper in a flask. The recovered filtrate solution was used for the determination of total phenolics content and DPPH percent inhibition of chevon balls.

The total phenolic content was estimated as per the method documented by Zhang et al. [13]. For the estimation of TPC, 0.3 mL of (0.20 N Folin–Ciocalteu’s phenol reagent) was blended with 0.60 mL sample extract to make a homogeneous solution. Finally, it was mixed with 2.4 mL of (20% Na_2_CO_3_) and kept in the dark for 10 min. After completion of incubation, the sample was filtered and absorbance was recorded at 730 nm. The DPPH percent inhibition of MSE added and control meatball was estimated as per the method given by Kato et al. [14]. In total, 1 mL of Tris-HCL (buffer 0.1 M and pH 7.4) was transferred in a test tube followed by the addition of 3.9 mL of DPPH regent (250 µM) 0.1 mL of the sample extract was added. The absorbance of the sample was recorded at t = 0 and t = 20 min.

### 2.5. Lipid Oxidation and Microbial Quality

The peroxide value (PV) of the chevon meatball was estimated as per the method given by Koniecko [15]. For analysis of PV, 2.5 g of sample and anhydrous Na_2_SO_4_ was mixed in 15 mL chloroform for 2 min. The mixed sample was filtered through Whatman filter (paper No. 1). In total, 12.5 mL of the above filtrate was transferred into a flask and blended with 15 mL of glacial acetic acid and, subsequently, 2 mL of saturated KI solution was poured in and allowed to rest for 2 min. After, that 50 mL of distilled water and 1 mL of (1%) starch solution were transferred into the same flask. Finally, the sample was titrated against (0.1 N) sodium thiosulfate until the endpoint. The thiobarbituric-acid-reactive substance (TBARS) content of the meatballs was assessed as per procedure by Witte et al. [16]. Serial dilution of the sample was prepared for the determination of the microbial quality of the product and was determined as per the methods given by APHA [17].

### 2.6. Sensory Analysis

Prior to the analysis of the samples, all sensory panelists were trained and selected on the basis of willingness to eat meat, availability and health condition. Sensory attributes were evaluated between 3:30 and 4: 30 PM. A panel of seven trained (both male and female) scientists and researchers in triplicate (n = 7 × 3 = 21) were selected and approved by the departmental research committee for assessing the sensory characteristics (color and appearance, flavor, texture and overall acceptability) of the meatball as per the method described by Keeton [18]. Color and appearance, flavor, texture and overall acceptability were evaluated on the basis of a 9-point hedonic scale, where the scale was rated as 1 = extremely poor, 5 = neither like nor dislike, and 9 = excellent. Coded chevon meatballs and drinking water were provided to each taster separately for the rinsing of the oral cavity during the analysis of the sample.

### 2.7. Statistical Analysis

The experiment was conducted three times and data were recorded twice for each attribute. Collected data were subjected to two-way ANOVA analysis (SPSS 22.0 (SPSS Inc., Chicago, IL, USA)) software and Duncun’s multiple range test as per the method given by Snedecor and Cochran [19].

## 3. Results and Discussion

### 3.1. Change in pH and Water Activity of Mango Seed Kernel Extract Incorporated Chevon Meatballs

The pH of the food products depends on the type of ingredients added in product formulation, microbial qualities and series of inevitable chemical reactions during the storage of foods. The pH and water activity of the chevon meatballs are presented (Table 2). The pH value varied considerably (*p* < 0.05) among the groups; however, during storage, the pH value decreased considerably (*p* < 0.05). The decreased pH value in control and treated groups could be due to microbial deterioration. It might be attributed to the vital activity of the lactic acid bacteria (LAB) which change carbohydrates into organic acids [20]. Capita et al. [21] also found a decrease in pH value from 6.39 to 6.16 during storage of goat patties. Similarly, Kumar et al. [3] also found a decrease in pH value from 6.16 to 5.39 during storage of pig meat patties. Water activity is an important parameter because it is directly related to the microbial quality of the meat products. The water activity content did not differ significantly (*p* > 0.05) up to the third day of storage; afterward, it varied significantly (*p* < 0.05) among groups throughout storage. Water activity decreased during storage among groups across storage. However, the water activity content in treated samples was much better maintained than in control, which might be due to the incorporation of different concentration of MSE that binds with hydrophilic groups and produces an osmoregulatory effect. The decrease in water activity might be due to the reduction in moisture content from products. Similarly, findings were also documented by Verma et al. [22] for meatballs during storage.

### 3.2. Change in Total Phenolics Content and DPPH (%) Inhibition of Mango Seed Kernel Extract Incorporated Chevon Meatballs

**Figure 1:**
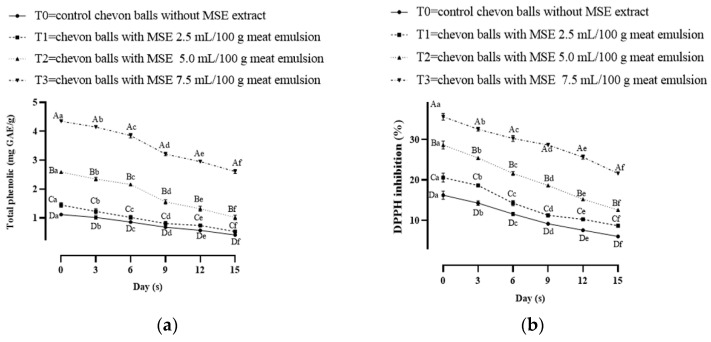
(**a**,**b**) Change total phenolics content and DPPH (%) inhibition of mango seed kernel extract incorporated chevon meatballs. T0 = control (without mango seed extract), T1 = (2.5 mL/100 g of meat emulsion *v*/*w*), T2 = (5.0 mL/100 g of meat emulsion *v*/*w*) and T3 = (7.5 mL/100 g of meat emulsion *v*/*w*). Mean values followed by different lower-case letters daywise and upper-case letters groupwise differ significantly (*p* < 0.05). n = 6.

Total phenolic content improved significantly (*p* < 0.05) as the concertation of MSE increased in the groups (Figure 1a). Lim et al. [23] stated that the presence of phyto-active compounds in mango seed kernel was responsible for total phenolic activity in a dose-dependent manner and a range from 18.19 to 101.68 mg GAE/g. Asif et al. [24] reported that mango kernel extract is a good source of gallic acid (6.0 mg/100 g) which acts as an antioxidant. Dey et al. [25] reported that phenolic groups have phenolic rings that can neutralize the free electron by donating hydrogen from hydroxyl groups, metal-binding potential and radical scavenging activities due to the presence of an aromatic ring. Decreasing trends of total phenolic content were noticed among all groups from the initial to the last days of storage, as expected. The reduction in the total phenolic content as storage time elapsed might be due to the decline in phenolic compounds as being used up in the process of neutralization/stabilization of lipid oxidation. Comparably, findings were also documented by Araujo et al. [26] for pig meat products. A 2,2-diphenylpicrylhydrazyl inhibition assay is one of the important parameters for the evaluation of the antioxidant activity of the phyto-active substance. The DPPH activity varied considerably (*p* < 0.05) in groups and increased in a concentration-dependent manner among treatments and decreased throughout storage (Figure 1b). The highest percent inhibition of DPPH was measured for the T3 groups and the lowest was for the control throughout storage. The antioxidant activity (TPC and DPPH) of mango seed kernel extract is also interrelated to the covalent bonds formed in the meat products during the processing and heating of products, the nature of bioactive compounds of MSE such as hydrophilicity/hydrophobicity and the structural configuration of active compounds. Ribeiro et al. [27] also found that mango seed extracts showed radical inhibition due to the presence of mangiferin. The antioxidant activity decrease might be due to the participation of these phyto-active compounds in the stabilization of free radicals formed during storage. Ergezer et al. [28] also reported a decrease in total phenolic content and DPPH percent inhibition during the storage study of the meat patties prepared with the addition of *Cynara scolymus* L. Extract.

### 3.3. Change in Lipid Oxidation of Mango Seed Kernel Extract Incorporated Chevon Meatballs

**Figure 2:**
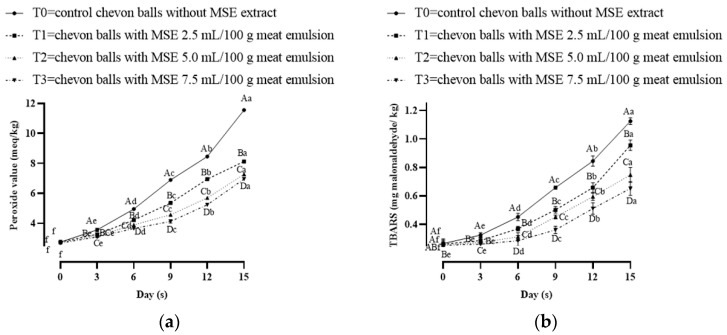
(**a**,**b**) Change in lipid oxidation of mango seed kernel extract incorporated chevon meatballs. T0 = control (without mango seed extract), T1 = (2.5 mL/100 g of meat emulsion *v*/*w*), T2 = (5.0 mL/100 g of meat emulsion *v*/*w*) and T3 = (7.5 mL/100 g of meat emulsion *v*/*w*). Mean values followed by different lower-case letters daywise and upper-case letters groupwise differ significantly (*p* < 0.05). n = 6.

Peroxide value is the primary oxidative product, whereas thiobarbituric-acid-reactive substance values indicate the end of the secondary lipid oxidation. These oxidative products also affect the sensorial characteristics of the food products which ultimately affect consumer perception. Peroxide value and thiobarbituric-acid-reactive substances (TBRAS) are important attributes and critically play an important role in assessing the shelf life of meat products. Figure 2a shows the consequence of the peroxide value analyses of the MSE-added chevon meatball and control over 15 days of refrigerated storage. On the initial day, PV did not differ significantly (*p* > 0.05) (2.68–2.75 meq/kg) among all groups, but it differed considerably (*p* < 0.05) among all samples from 3 to 15 days. The control had the highest PV values and T3 had the lowest PV value throughout storage. During storage, PV of all treatments including control increased significantly (*p* < 0.05) across the entire storage period, but the rate of increment in MSE treated groups was considerably lower in a concentration-dependent manner. Choe et al. [29], contemplating the effect of persimmon peel (*Diospyros kaki Thumb*.) extracts on fat pig meat stored at 4 ± 1 °C, reported similar results. However, the TBARS number differed considerably (*p* < 0.05) from 0 to 15 days of storage among all groups (Figure 2b). The TBARS number also increased during storage but the rate of lipid oxidation was comparatively lower in MSE-extract-added groups, as expected. The addition of MSE to the chevon balls lowered PV and TBARS values compared to the controls. Mango seed kernel is rich in antioxidants with high bioactivity compounds like phenols carotenoids and flavonoids. The presence of these compounds in MSE may effectively reduce the rate of oxidation of lipid and protein molecules via a reduction in the generation of free radical/scavenging free radicals resulting in an enhanced shelf life of food products. Similar findings were also reported by Singh et al. [30] for goat sorpotel prepared by the addition of finger millet. Minatel et al. [31] stated that phenolic compounds have the capacity to stabilize free radicals by donating electrons or hydrogen atoms, as well as sequestering metals. Phenolic compounds also have the capability to decrease the production of hydroperoxide compounds from oxidative deterioration of lipids [32]. Similar results were also reported by Abdel-Moemin [33] for lower TBARS (6–6.25) values in beef sausages prepared with the addition of mango seed kernel than control.

### 3.4. Change in Microbial Quality of Mango Seed Kernel Extract Incorporated Chevon Meatballs

The microbiological quality of chevon meatballs prepared with the addition of mango seed kernel extract and control are depicted in (Table 3). The standard plate count of the chevon meatballs varied significantly (*p* < 0.05) among the groups throughout storage except for the initial days. The standard plate counts increased during storage, but significantly (*p* < 0.05) lower counts were observed in mango seed kernel extract groups than in control groups. The standard plate count values for T0, T1, T2 and T3 at the end of the storage were 6.14, 5.96, 5.19 and 5.58 CFU/g, respectively. Various researchers have also reported that the standard plate counts increased throughout storage in goat meat nuggets [2] and in chevon sorpotel [30]. Psychrophilic counts are an important microbial count for the assessment of the microbial quality of products stored at refrigerated temperatures. Psychrophilic counts were observed on the ninth day in control and T1 during the storage study. Psychrophilic counts differed significantly (*p* < 0.05) among all groups at the 12th and 15th days of storage. However, its counts were significantly (*p* < 0.05) lower in T3 followed by T2, T1 and control. The colonies of yeast and mold were not observed until the ninth day of storage among all groups. Gómez-Maldonado et al. [34] attributed antimicrobial activity to the presence of gallates, gallotannins and proanthocyanidins. Huang et al. [35] reported that MSE extract was more effective against Gram-positive bacteria than Gram-negative. Kanatt and Chawla [10] also reported that the addition of mango extract exhibited antimicrobial activity against various foodborne microbes in chicken meat during storage. Psychrophilic counts were absent among all the groups up to the sixth day of storage, and they were noticed first on day 9 in the control and T1 groups, on day 12 in the T2 group, and on day 15 in the T3 group. It might be due to the slow release of antimicrobial phyto-active compounds storage. Alaiya et al. [36] reported that the antimicrobial activity of mango seed extract is due to the presence of gallotannin and it has the capacity to bind iron and destabilization of membrane-bound proteins. Engels et al. [37] also reported the antimicrobial effect of mango seed kernel against various food spoilage microorganisms. Yeast and mold counts were not detected among all groups on the ninth day of the storage period which might be due to the antifungal activity of mango seed kernel extract. Gómez-Maldonado et al. [34] also reported the antifungal activity of mango seed kernel due to presence of mangiferin, ferulic acid, rutin and quercetin.

### 3.5. Change in Sensory Quality of Mango Seed Kernel Extract Incorporated Chevon Meatballs

Sensory attributes of any food products are an important parameter for the consumer’s perception. However, the sensory attributes of the food products are directly related to microbial quality and lipid and protein oxidation. Chevon meatballs were evaluated for sensory characteristics like color and appearance, texture, flavor and overall acceptability, as depicted in Table 4. Color and appearance did not differ considerably (*p* > 0.05) among all groups at day 0 of storage, but differed significantly from day 3 to 15 of storage. Sensory panelists rated lower scores for color and appearance as the storage period increased, which might be due to loss of moisture and formation of some metabolite of microbial growth and lipid oxidation. The decrease in color score might be due to the non-enzymatic reaction between the oxidative products and free amino acid of meat. The flavor of the meat products is an important attribute of the meat products and it was rated as a lower score by sensory panelists as storage time increased. The reduction in flavor score might have been influenced by increase in the amount of peroxide value, aldehyde, ketones, dienes and free fatty content in the stored products [38]. Similarly, Ref. [39] also reported that formation these compounds in meat products during storage leads to decrease in flavor score of meat products. The texture of the chevon meatballs prepared with the incorporation of MSE was maintained better than the control during the storage, which might be due to lower deteriorative change. Textural quality of chevon meatballs did not differ significantly (*p* > 0.05) up to the third day of storage, but differed (*p* < 0.05) from the sixth day to the end of storage. Sensory panelists rated lower scores for all groups during storage, which might be due to the decrease in the textural quality of chevon meatballs. Oxidation and microbial deterioration play a major role in the deterioration of the textural quality of products. The decrease in the textural attributes from 7.18 to 5.92 attributed to lipid oxidation and microbial deterioration was also reported by Kumar et al. [3] for meat patties. Overall, the acceptability of meatballs also followed a declining trend during entire storage which might be due to oxidative and microbial deterioration, though all attributes were better maintained in treated groups than control. The overall acceptability scores were rated higher by the sensory panelist for T3 (6.54) followed by T2 (6.33), T1 (6.23) and T0 (6.06), respectively. Similarly, Abdel-Moemin [33] also reported a decrease in sensory attributes of beef sausages during storage at lower rate in mango-seed-kernel-added products than in the control. Pinsirodom et al. [40] reported that the addition of mango seed extract (6.10 at end of storage) to fish meat maintained better sensory attributes than the control (5.85 at the end of storage) due to lower lipid and protein oxidation.

## 4. Conclusions

It can be concluded that incorporation of MSE at 7.5 mL/100 g (T3) in chevon meatballs resulted in significantly better maintained oxidative stability as evaluated in terms of lipid peroxidation, microbiological qualities and sensory characteristics. The retardation of the formation of PV, TBARS and FFA ensured better maintained qualities of chevon balls in terms of sensory quality in T3 groups than other groups. Mango seed kernel is a good source of phyto-preservatives and was found to be an excellent additive to better maintain the shelf life of chevon meatballs (T3) for up to 15 days at refrigeration temperature. In order to improve the findings in the future, experiments must be conducted with the aim of recognizing the phyto-active compounds in the mango kernel seed extracts which contribute to the antioxidant and antimicrobial activity. Also, it may be recommended for the evaluation of any toxicity-related issue of MSE extract in vitro or in vivo.

## Figures and Tables

**Table 1:** Formulation of chevon balls.

Ingredients (%)	T0	T1	T2	T3
Meat	68.20	68.20	68.20	68.20
Refined oil	6.0	6.0	6.0	6.0
Chilled water	10.0	7.5	5.0	2.5
Egg yolk	3.0	3.0	3.0	3.0
Salt	1.5	1.5	1.5	1.5
Spice mix.	2.0	2.0	2.0	2.0
Gram dal	3.0	3.0	3.0	3.0
Condiments	3.0	3.0	3.0	3.0
Hydrated soya chunk (1 soya:3 water)	3.0	3.0	3.0	3.0
Sodium tetra pyro-phosphate (STPP)	0.3	0.3	0.3	0.3
MSE extract (*v*/*w*)	0.0	2.5	5.0	7.5
Nitrite (ppm)	120	120	120	120

T0 = control (without mango seed extract), T1 = (2.5 mL/100 g of meat emulsion *v*/*w*), T2 = (5.0 mL/100 g of meat emulsion *v*/*w*), and T3 = (7.5 mL/100 g of meat emulsion *v*/*w*).

**Table 2:** Change in pH and water activity of mango-seed-kernel-extract-incorporated chevon meatballs.

Groups	Day 0	Day 3	Day 6	Day 9	Day 12	Day 15	Groups	Days	Groups × Days
pH									
T0	6.26 ± 0.05 ^Ba^	6.20 ± 0.06 ^Bb^	6.19 ± 0.03 ^Cb^	6.14 ± 0.02 ^Cc^	6.10 ± 0.03 ^Ccd^	6.04 ± 0.04 ^Cd^	*	*	NS
T1	6.28 ± 0.03 ^Ba^	6.26 ± 0.03 ^ABa^	6.24 ± 0.02 ^Bab^	6.20 ± 0.04 ^Bb^	6.17 ± 0.05 ^Bbc^	6.15 ± 0.03 ^Bc^			
T2	6.32 ± 0.02 ^Aa^	6.29 ± 0.04 ^Aa^	6.27 ± 0.01 ^ABab^	6.25 ± 0.03 ^Ab^	6.21 ± 0.04 ^ABc^	6.19 ± 0.05 ^Ac^			
T3	6.35 ± 0.04 ^Aa^	6.31 ± 0.05 ^Aab^	6.30 ± 0.03 ^Aab^	6.28 ± 0.05 ^Ab^	6.24 ± 0.03 ^Ac^	6.20 ± 0.02 ^Ad^			
Water activity (a_W_)									
T0	0.875 ± 0.003 ^a^	0.865 ± 0.005 ^b^	0.852 ± 0.002 ^Bc^	0.841 ± 0.002 ^Bd^	0.835 ± 0.003 ^Be^	0.821 ± 0.004 ^Cf^	*	*	NS
T1	0.871 ± 0.004 ^a^	0.869 ± 0.004 ^a^	0.851 ± 0.005 ^Bb^	0.838 ± 0.004 ^Bc^	0.832 ± 0.006 ^Bd^	0.826 ± 0.005 ^Ce^			
T2	0.874 ± 0.005 ^a^	0.871 ± 0.006 ^a^	0.862 ± 0.006 ^Ab^	0.855 ± 0.005 ^Ac^	0.841 ± 0.002 ^Ad^	0.831 ± 0.002 ^Be^			
T3	0.876 ± 0.002 ^a^	0.870 ± 0.003 ^b^	0.866 ± 0.003 ^Ac^	0.859 ± 0.007 ^Ad^	0.846 ± 0.005 ^Ae^	0.838 ± 0.003 ^Af^			

T0 = control (without mango seed extract), T1 = (2.5 mL/100 g of meat emulsion *v*/*w*), T2 = (5.0 mL/100 g of meat emulsion *v*/*w*) and T3 = (7.5 mL/100 g of meat emulsion *v*/*w*). Mean values followed by different lower-case letters daywise and upper-case letters groupwise differ significantly (*p* < 0.05). n = 6. * = significant and NS = non-significant.

**Table 3:** Change in microbial quality of mango seed kernel extract incorporated chevon meatballs.

Groups	Day 0	Day 3	Day 6	Day 9	Day 12	Day 15	Groups	Days	Groups × Days
Standard Plate Counts (CFU/g)									
T0	2.51 ± 0.12 ^f^	2.89 ± 0.13 ^Ae^	3.56 ± 0.09 ^Ad^	4.26 ± 0.08 ^Ac^	5.12 ± 0.07 ^Ab^	6.14 ± 0.09 ^Aa^	*	*	*
T1	2.45 ± 0.09 ^f^	2.68 ± 0.15 ^ABe^	3.12 ± 0.08 ^Bd^	3.96 ± 0.07 ^Bc^	4.51 ± 0.11 ^Bb^	5.96 ± 0.06 ^Ba^			
T2	2.38 ± 0.13 ^e^	2.56 ± 0.14 ^Be^	2.96 ± 0.07 ^BCd^	3.65 ± 0.12 ^Cc^	4.12 ± 0.10 ^Cb^	5.19 ± 0.07 ^Ca^			
T3	2.40 ± 0.14 ^e^	2.51 ± 0.11 ^Be^	2.89 ± 0.12 ^Cd^	3.45 ± 0.10 ^Cc^	3.85 ± 0.09 ^Db^	4.58 ± 0.10 ^Da^			
**Psychrophilic counts (CFU/g)**									
T0	ND	ND	ND	1.18 ± 0.06 ^c^	1.48 ± 0.10 ^Ab^	1.98 ± 0.08 ^Aa^	*	*	NS
T1	ND	ND	ND	1.15 ± 0.08 ^c^	1.32 ± 0.13 ^ABb^	1.65 ± 0.11 ^Ba^			
T2	ND	ND	ND	ND	1.15 ± 0.09 ^Bb^	1.48 ± 0.12 ^BCa^			
T3	ND	ND	ND	ND	ND	1.36 ± 0.08 ^C^			
**Yeast and Mould counts (CFU/g)**									
T0	ND	ND	ND	ND	1.43 ±0.06 ^Ab^	1.71 ± 0.10 ^Aa^	*	*	NS
T1	ND	ND	ND	ND	1.27 ± 0.05 ^Bb^	1.56 ± 0.08 ^Ba^			
T2	ND	ND	ND	ND	1.36 ± 0.07 ^ABb^	1.51 ± 0.09 ^Ba^			
T3	ND	ND	ND	ND	1.12 ± 0.06 ^Cb^	1.43 ± 0.10 ^Ba^			

T0 = control (without mango seed extract), T1 = (2.5 mL/100 g of meat emulsion *v*/*w*), T2 = (5.0 mL/100 g of meat emulsion *v*/*w*) and T3 = (7.5 mL/100 g of meat emulsion *v*/*w*). Mean values followed by different lower-case letters daywise and upper-case letters groupwise differ significantly (*p* < 0.05). n = 6. * = significant, NS = non-significant and ND = not detected.

**Table 4:** Change in sensory quality of mango seed kernel extract incorporated chevon meatballs.

Groups	Day 0	Day 3	Day 6	Day 9	Day 12	Day 15	Groups	Days	Groups × Days
Color and Appearance									
T0	8.21 ± 0.11 ^a^	7.85 ± 0.08 ^Bb^	7.24 ± 0.07 ^Cc^	6.89 ± 0.13 ^Cd^	6.24 ± 0.09 ^Ce^	5.96 ± 0.08 ^Cf^	*	*	*
T1	8.15 ± 0.09 ^a^	7.79 ± 0.06 ^Bb^	7.45 ± 0.11 ^Bc^	7.12 ± 0.08 ^Bd^	6.65 ± 0.08 ^Be^	6.23 ± 0.12 ^Bf^			
T2	8.22 ± 0.08 ^a^	8.12 ± 0.12 ^Ab^	7.64 ± 0.09 ^ABc^	7.21 ± 0.07 ^Ad^	6.96 ± 0.12 ^Ae^	6.34 ± 0.13 ^Bf^			
T3	8.18 ± 0.012 ^a^	8.08 ± 0.11 ^Ab^	7.76 ± 0.12 ^Ac^	7.35 ± 0.11 ^Ad^	7.02 ± 0.13 ^Ae^	6.85 ± 0.10 ^Af^			
**Flavor**									
T0	8.31 ± 0.11 ^a^	8.12 ± 0.12 ^b^	7.56 ± 0.06 ^Bc^	7.02 ± 0.11 ^Cd^	6.45 ± 0.08 ^Ce^	6.08 ± 0.12 ^Cf^	*	*	*
T1	8.25 ± 0.13 ^a^	8.15 ± 0.09 ^b^	7.79 ± 0.10 ^Ac^	7.35 ± 0.08 ^Bd^	6.76 ± 0.11 ^Be^	6.21 ± 0.07 ^BCf^			
T2	8.18 ± 0.09 ^a^	8.09 ± 0.06 ^a^	7.81 ± 0.09 ^Ab^	7.48 ± 0.12 ^Bc^	6.98 ± 0.13 ^Bd^	6.33 ± 0.08 ^Be^			
T3	8.22 ± 0.07 ^a^	8.11 ± 0.013 ^a^	7.86 ± 0.08 ^Ab^	7.64 ± 0.06 ^Ac^	7.12 ± 0.06 ^Ad^	6.74 ± 0.06 ^Ae^			
**Texture**									
T0	8.33 ± 0.14 ^a^	8.18 ± 0.12 ^b^	7.41 ± 0.13 ^Cc^	7.12 ± 0.11 ^Cd^	6.66 ± 0.12 ^Ce^	6.13 ± 0.09 ^Cf^	*	*	NS
T1	8.25 ± 0.11 ^a^	8.16 ± 0.15 ^a^	7.62 ± 0.11 ^Bb^	7.21 ± 0.09 ^BCc^	6.84 ± 0.06 ^Bd^	6.28 ± 0.08 ^Be^			
T2	8.21 ± 0.13 ^a^	8.11 ± 0.09 ^a^	7.74 ± 0.08 ^ABb^	7.42 ± 0.12 ^Ac^	6.91 ± 0.11 ^ABd^	6.32 ± 0.12 ^Be^			
T3	8.28 ± 0.10 ^a^	8.15 ± 0.07 ^a^	7.89 ± 0.09 ^Ab^	7.36 ± 0.07 ^Ac^	7.16 ± 0.13 ^Ad^	6.67 ± 0.10 ^Ae^			
**Overall acceptability**									
T0	8.26 ± 0.08 ^a^	8.02 ± 0.08 ^b^	7.47 ± 0.10 ^Bc^	7.06 ± 0.09 ^Cd^	6.58 ± 0.06 ^Ce^	6.06 ± 0.07 ^Cf^	*	*	NS
T1	8.23 ± 0.06 ^a^	8.10 ± 0.12 ^b^	7.67 ± 0.13 ^ABc^	7.13 ± 0.12 ^BCd^	6.71 ± 0.11 ^Be^	6.23 ± 0.12 ^Bf^			
T2	8.21 ± 0.09 ^a^	8.12 ± 0.14 ^a^	7.82 ± 0.08 ^Ab^	7.21 ± 0.11 ^Bc^	6.84 ± 0.14 ^Bd^	6.33 ± 0.06 ^Be^			
T3	8.18 ± 0.10 ^a^	8.06 ± 0.09 ^a^	7.88 ± 0.11 ^Ab^	7.54 ± 0.08 ^Ac^	7.09 ± 0.12 ^Ad^	6.54 ± 0.09 ^Ae^			

T0 = control (without mango seed extract), T1 = (2.5 mL/100 g of meat emulsion *v*/*w*), T2 = (5.0 mL/100 g of meat emulsion *v*/*w*) and T3 = (7.5 mL/100 g of meat emulsion *v*/*w*). Mean values followed by different lower-case letters daywise and upper-case letters groupwise differ significantly (*p* < 0.05). n = 27. * = significant and NS = non-significant.

## Data Availability

The original contributions presented in the study are included in the article, further inquiries can be directed to the corresponding author.
